# Fabry–Perot interferometric calibration of van der Waals material-based nanomechanical resonators†

**DOI:** 10.1039/d1na00794g

**Published:** 2021-11-23

**Authors:** Myrron Albert Callera Aguila, Joshoua Condicion Esmenda, Jyh-Yang Wang, Teik-Hui Lee, Chi-Yuan Yang, Kung-Hsuan Lin, Kuei-Shu Chang-Liao, Sergey Kafanov, Yuri A. Pashkin, Chii-Dong Chen

**Affiliations:** National Tsing Hua University Hsinchu 30013 Taiwan maguila@gate.sinica.edu.tw; Nano Science and Technology Program, Taiwan International Graduate Program, Academia Sinica and National Tsing Hua University Taiwan; Institute of Physics, Academia Sinica Nangang 11529 Taiwan chiidong@phys.sinica.edu.tw; Department of Physics, Lancaster University Lancaster LA1 4YB UK

## Abstract

One of the challenges in integrating nanomechanical resonators made from van der Waals materials in optoelectromechanical technologies is characterizing their dynamic properties from vibrational displacement. Multiple calibration schemes using optical interferometry have tackled this challenge. However, these techniques are limited only to optically thin resonators with an optimal vacuum gap height and substrate for interferometric detection. Here, we address this limitation by implementing a modeling-based approach *via* multilayer thin-film interference for *in situ*, non-invasive determination of the resonator thickness, gap height, and motional amplitude. This method is demonstrated on niobium diselenide drumheads that are electromotively driven in their linear regime of motion. The laser scanning confocal configuration enables a resolution of hundreds of picometers in motional amplitude for circular and elliptical devices. The measured thickness and spacer height, determined to be in the order of tens and hundreds of nanometers, respectively, are in excellent agreement with profilometric measurements. Moreover, the transduction factor estimated from our method agrees with the result of other studies that resolved Brownian motion. This characterization method, which applies to both flexural and acoustic wave nanomechanical resonators, is robust because of its scalability to thickness and gap height, and any form of reflecting substrate.

## Abstract 2

Multilayer interference approach is applied to confocal microscopy images of an electromotive-driven van der Waals material nanomechanical resonator to extract its thickness, gap height, and motional amplitude in the frequency and spatial domains.

## Graphical abstract

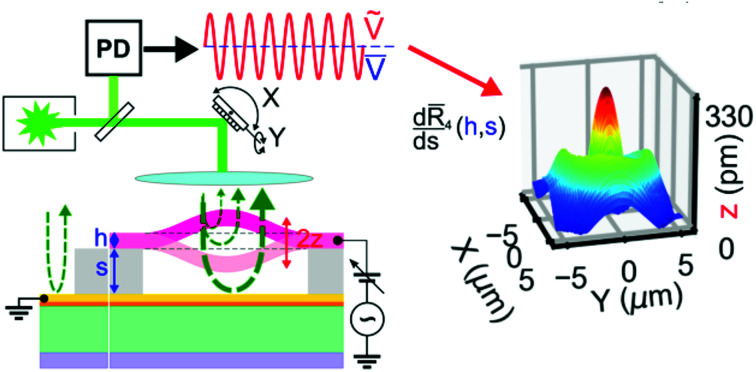

Nanomechanical resonators (NMRs) made from van der Waals materials like graphene^1–3^ and transition metal dichalcogenides^4–7^ offer plausible platforms for measuring minuscule forces^3,8^ and ultralight masses^9^ with unprecedented sensitivity and motion responsivity^5,7,10^ to external stimuli. Typical NMR-based sensing platforms involve monitoring changes in features of the driven frequency response, primarily the shifts of the mechanical frequency and variations of the linewidth, in the presence of an actuating force.^11^ The calibrated vibrational amplitude at resonance, together with the resonator frequency, provides direct quantification of the Young's elastic modulus, effective mass, and driving force felt by the NMR from a single response spectrum.^12^ From a practical standpoint, monitoring the vibrational amplitude is essential for avoiding dynamic pull-in instability^13^ when driving the NMR at high amplitude, which limits the actuation ranges for electromotively driven NMRs. Furthermore, the vibrational resonant amplitude enables understanding of the nonlinear regime of motion^14,15^ and optomechanical effects on driven mechanics,^8,16,17^ both of which are accessible for mechanical resonators with nanoscale dimensions, and embedded in motion-sensitive interferometric platforms.

Calibration of the motional amplitude of NMRs using Fabry–Perot (FP) interferometry has been challenging because it requires pre-determination of device parameters such as thickness and spacer gap, which are difficult to ascertain and vary from device to device. Previous attempts of this calibration were shown through the resolving of the Brownian motion,^7,18,19^ analyzing the high-amplitude Duffing response,^3^ measuring the driven motion at higher harmonics,^20^ and monitoring photodetector responses.^12^ The calibration methods used in these previous studies, however, rely on specific device conditions such as optically transparent cross-sections,^2,7^ access to high amplitude nonlinear motion, and high optical-to-displacement responsivities^10^ from an optimal vacuum gap. These characteristics are specific to monolayer, bilayer, trilayer, and few-layer van der Waals materials, whose unique optical characteristics, ultralight mass, and mechanics more closely resemble near-transparent, prestressed membranes. The methods would not be accurate if one or several of these criteria are not met.

Also, there are applications where a smaller vacuum gap between the NMR and the substrate has advantages over the optimal gap for interferometric detection. These include large frequency tunability in electromotively driven NMRs,^9,21^ stronger electromechanical coupling between NMR and microwave cavities,^8,16^ and introducing optomechanics in an electromechanical system.^22–24^ For these applications, van der Waals materials of intermediate thickness (between 10 L and 100 L) offer comparable (if not better) optical reflectance-to-displacement responsivity^10^ and electromechanical coupling,^8,14,16,25^ and near strain-free mechanical frequencies^26^ as compared to their few-layer counterparts. Furthermore, they are easier to prepare *via* micromechanical exfoliation, and polymeric contaminants have a negligible contribution to their masses.

In this work, we show that the motion of an NMR can be calibrated by considering multilayer wave interference occurring on FP structures. To demonstrate the robustness of the technique, a thick van der Waals material, 2H-NbSe_2_, is used as the drumhead. 2H-NbSe_2_, at room temperature, is a semi-metallic material, whose number of layers, when increased, is difficult to differentiate with Raman spectroscopy signatures^27^ from the bulk, unlike MoS_2_.^28^ This material makes it a unique model for assessing the number of layers based on the layer-dependent refractive index. Our approach, implemented with laser scanning confocal microscopy, allows robust, non-contact and *in situ* determination of the layer thickness, spacer height, and device responsivity of each translucent flexible mirror. Our calibration scheme, using a 532 nm laser wavelength, reveals a subnanometer displacement response of NMRs with thickness exceeding 50 nm. Furthermore, calibrated spatial imaging of the driven fundamental mode of circular and elliptical NbSe_2_ drums enables direct investigation of the modal properties (*i.e.* effective mass and Young's elastic modulus) of the drumheads.

## Experimental

### Device fabrication

P-type doped Si chips (7 mm × 7 mm × 0.65 mm), with a thermally grown 543 nm thick SiO_2_ layer, were lithographically patterned with 20 nm Cr and 40 nm Au electrodes. The chips are cleaned, and then spin-coated with a CSAR-62 electron-beam resist to create the spacer. The resist is exposed in an electron-beam writer with drum holes and contact window patterns. After the development, the resist is baked at 180 °C for 9 minutes to harden the spacer. The spacer thickness, as measured with a commercial stylus profilometer, is 295 ± 10 nm. Bulk NbSe_2_ flakes purchased from HQ Graphene are exfoliated and transferred onto the patterned drum and contact windows using a dry polydimethylsiloxane stamp transfer process. Additional details on the flake transfer process are narrated in other studies.^29,30^

### Electromotive actuation and interferometric detection

The sample is placed in a vacuum box at ambient temperature (20–22 °C), with optical and electrical access, which is evacuated to a base pressure of 6 × 10^−7^ mbar to minimize viscous losses. The NbSe_2_ flake is galvanically connected to the output of the function generator, which creates DC and AC voltages between the flake and the grounded Au/Cr electrode forming a parallel-plate capacitor. This voltage produces an attractive force between the capacitor plates, which actuates the drums when the driving frequency matches the resonance frequency. The drums experience an electromotive force arising from the DC and AC voltage inputs of the function generator. A custom-built confocal microscope, with a continuous wave 532 nm laser (Lighthouse Photonics Sprout-G) and a spot size of roughly 1.9 μm, probes the out-of-plane position and motional amplitude of the drums interferometrically. A fast avalanche photodetector (Thorlabs APD130A/M) measures both the average and modulated intensities of light reflected from the drums, and converts them to the output voltages *V̄* and *Ṽ*. The data acquisition (DAQ) system records *V̄* at coordinates of *X* and *Y*. A high-frequency lock-in amplifier (Stanford Research SRS-844) processes *Ṽ* with the signal frequency referenced from the function generator. See Fig. S1† for a detailed schematic diagram of the interferometer setup.

## Results and discussion

### Determination of the thickness and vacuum gap *via* optical contrast

**Fig. 1:**
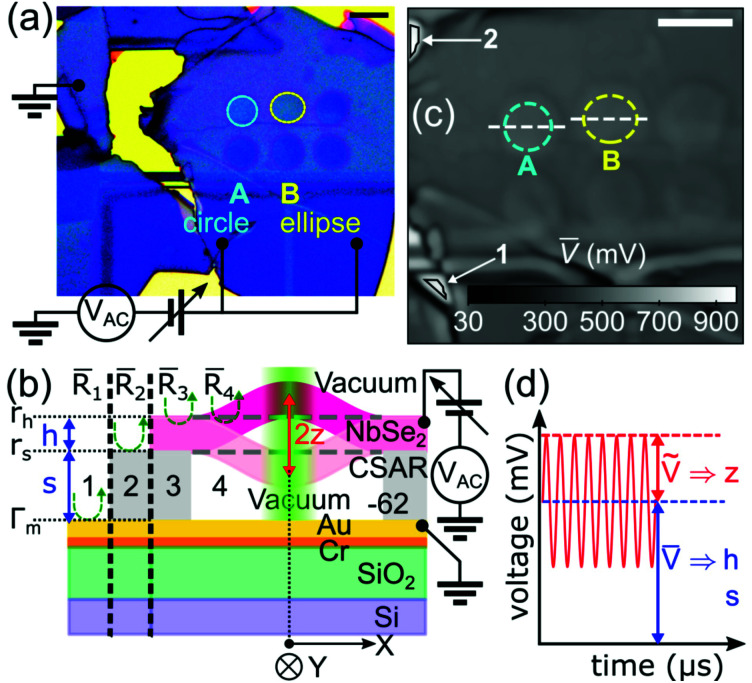
(a) Optical micrograph of the NbSe_2_ plate resonator devices. The circuit diagram shows the electromotive scheme used in driving the resonator. (b) Schematic drawing of the optical cross-section of the device (zones 3 and 4) and the references (zones 1 and 2) as measured *via* Fabry–Perot interference. The actuation circuit is added for clarity. (c) Confocal image showing devices A and B as scanned with a probe laser beam with wavelength *λ* = 532 nm. Scale bars in (a) and (c) are set to 10 μm. (d) Sketch of the output voltage of the fast photodetector *versus* time of a driven flexural resonator.

Fig. 1(a) shows device A, a circular drum with a hole diameter of 7 μm, and device B, an elliptical drum with hole diameters of 8 μm (*X*, major axis) and 7 μm (*Y*, minor axis). The devices share the same flake, ground electrode, and driving voltages. The NbSe_2_ flake and ground electrodes are separated by an insulating layer (electron-beam resist CSAR-62) and vacuum spacers, and hence form Fabry–Perot cavities for detection, and capacitors for actuation. A large rectangular opening, located tens of microns below the drum centers, allows the flake to connect to the voltage-controlled Au/Cr electrodes. The motion of the electromotively driven drumheads is detected interferometrically in a high vacuum environment.

Our method relies on different contrasts of light elastically reflected from each zone, as shown in Fig. 1(b). The flake (pink bar) acts as a translucent movable mirror with thickness *h*, which is separated from the ground electrode by a spacer of height *s*. For convenience, the reflected intensity is expressed in terms of reflectance *R*, which is the ratio of the total reflected light intensity to the incident intensity. Stationary mirrors have only DC component *R* = *R̄*, while movable mirrors have both *R̄* and AC component *R̃*. Zones 1 and 2 represent two stationary mirrors: stacks of gold, orange, green and blue bars having reflectance *R*_1_ = *R̄*_1_ and a mirror covered with a spacer (light gray) having reflectance *R*_2_ = *R̄*_2_, respectively. Zone 3 represents two stationary mirrors separated by a dielectric gap (clamp) with reflectance *R*_3_ = *R̄*_3_. Finally, zone 4 is the main FP cavity composed of one stationary and one movable mirror, which are separated by a vacuum gap with reflectance *R*_4_. Here, zones 1 and 2 are references for zones 4 and 3, respectively. Scanning mirrors in the measurement setup move the laser spot in each zone a distance *X* and *Y* away from the center of the drums.

Application of DC and AC voltages to the flake exerts an attractive force; the NMR responds with an out-of-plane motional amplitude *z* at a driving frequency *f*_d_. Due to the position and motion of the movable mirror in zone 4, the main FP cavity has reflectance *R*_4_ = *R̄*_4_ + *R̃*_4_(*f*_d_), with *R̄*_4_ ≫ *R̃*_4_(*f*_d_). Fig. 1(d) shows the photodetector output signal *V* acquired from *R*_4_. Both the DC component *V̄* and the AC component *Ṽ* of the output signal are proportional to *R̄*_4_ and *R̃*_4_, respectively. Amplitude *z* is determined after obtaining *h* and *s*.

Though we calculate *R̄*_1–4_ using the multilayer interference approach^31–33^ (MIA), the reflectance of FP cavities with four interfaces^34^*R̄*_3,4_ captures the stationary reflections occurring for each drum. Here, we assume that the coherent probe light, having wavelength *λ*, originates from a point source and propagates from a semi-infinite vacuum layer. The drum and the bottom mirror have complex refractive indices *n̂*_h_ (ref. 27) and *n̂*_m_, respectively, while the spacers have real refractive index *n̂*_s_ (*n̂*_s,drum_ for the vacuum spacer and *n̂*_s,clamp_ for the CSAR-62 spacer). In this geometry, the vacuum–NMR, NMR–spacer, and spacer–mirror interfaces contribute significantly to the cavity's overall reflectance. The total reflectance is defined as1
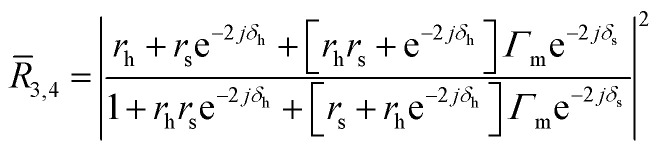
where *δ*_h_ = 2π*n̂*_h_*h*/*λ* is the optical phase thickness of the NMR, *δ*_s_ = 2π*n̂*_s_*s*/*λ* is the optical phase thickness of the spacer, *r*_h_ = (1 − *n̂*_h_)/(1 + *n̂*_h_) is the Fresnel coefficient of the vacuum–NMR interface, *r*_s_ = (*n̂*_h_ − *n̂*_s_)/(*n̂*_h_ + *n̂*_s_) is the Fresnel coefficient of the NMR–spacer interface, and *Γ*_m_ = (*n̂*_s_ − *n̂*_m_)/(*n̂*_s_ + *n̂*_m_) is the equivalent Fresnel coefficient of the spacer–mirror interface. For convenience, *Γ*_m_ is computed using MIA.†

Fig. 1(c) shows the confocal image constructed from the DC voltage *V̄* of the photodetector. The image reveals the topographical features of the drum and its surroundings. The measured voltages that correspond to zones 1 and 2, mentioned in Fig. 1(b), are taken from the areas shown with the arrows in Fig. 1(c) and they are represented by *V̄*_1_ and *V̄*_2_, respectively. Following the white dashed line in Fig. 1(c), the measured voltage outside the boundary is defined by *V̄*_3_, while the measured voltage inside is represented by *V̄*_4_. It is important to note that the mismatch between the drum boundaries in Fig. 1(a) and (c) is caused by the deformation of the edge of the drum holes during the elastomeric stamp step of the flake transfer.

Since *R̄* is susceptible to scattering losses,^35^ we circumvent this issue by normalizing the Michelson contrast^34,36^ of each FP cavity to its reference. Having defined the experimental and calculated reflectance, the cavity's optical contrast, *C*, is quantified as *C* = (*R̄*_3,4_ − *R̄*_2,1_)/(*R̄*_3,4_ + *R̄*_2,1_), where *R̄*_3,4_ is the stationary reflectance of the FP cavity, and *R̄*_2,1_ is the stationary reflectance of the cavity's reference. Apparently, *C* ranges between −1 and 1, with zero denoting no difference from the reference. If *C* is positive, then the cavity is brighter than the reference. Otherwise, the cavity is darker than its reference.

**Fig. 2:**
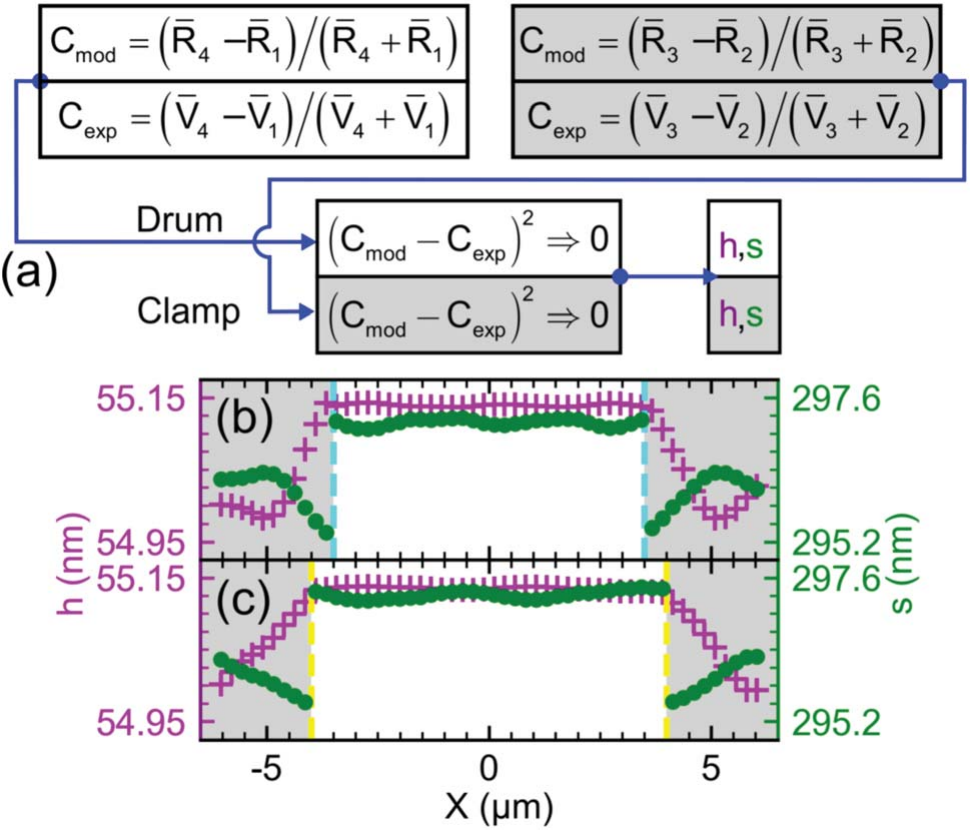
(a) Diagram for determining *h* and *s* for the clamp and drum zones. Minimization of the difference between the experimental contrast (*C*_exp_) and the modelled contrast (*C*_mod_) results in *h* and *s* profiles for device A (b) and device B (c). Colored dashed lines refer to the hole radius set in Fig. 1(a and c), separating the drum (white fills) and clamp (gray fills) zones.

The output voltages measured for each pixel along the dashed lines in Fig. 1(c) are converted into contrast values for devices A and B, as depicted in Fig. 2(a). The experimental contrast *C*_exp_ represents the ratio of voltages acquired from different zones in the confocal image of each device while the modelled contrast *C*_mod_ is derived using MIA.† See Fig. S5† for the correspondence between the modelled contrast and its *h* and *s* pairs for devices A and B. Fig. 2(b and c) show the resulting *h* and *s* cross-sectional profiles acquired from minimizing the difference between the experimental contrast values and the contrasts generated by MIA. The mean resonator thicknesses and spacer heights are in excellent agreement with the mean values listed in Table 1. The thickness of the drums measured by our method agrees well with atomic force microscopy measurements for different areas of the flake, as shown in Fig. S2.† The spacer height for both drums and clamps agrees well with the stylus profilometer measurements. From the flake thickness of about 55 nm, we deduce 92 layers of NbSe_2_ sheets assuming a single layer thickness of 0.6 nm.^37^

**Table 1:** Mean flake and spacer thicknesses of the NbSe_2_ drum and clamp zones

Devices	A	B
*h*_drum_ (nm)	55.139 ± 0.002	55.135 ± 0.002
*h*_clamp_ (nm)	55.03 ± 0.05	55.05 ± 0.04
*s*_drum_ (nm)	297.2 ± 0.1	297.3 ± 0.1
*s*_clamp_ (nm)	296.0 ± 0.3	295.9 ± 0.3

The *h* profiles in Fig. 2(b and c) show a hundred picometer variation between the drum and clamp zones. Meanwhile, buckling is observed in the *s* profiles in Fig. 2(b and c) as *s*_drum_ for both devices is greater than *s*_clamp_ by 1.2–1.4 nm. We see the drumheads bulge^38–40^ presumably due to the pressure difference between the trapped air in the drum hole and the vacuum environment. The surface roughness of the movable mirror likely originates from the thermally grown oxide^41^ on the surface of the stationary mirror.

### Conversion of optical signals to motional amplitudes

Having determined the mean *h*_drum_ and *s*_drum_, we evaluate the optical reflectance-to-displacement responsivity |d*R̄*_4_/d*s*| of each drum. This quantity is obtained from *R̃*_4_(*f*_d_) = |d*R̄*_4_/d*s*|*z*(*f*_d_). The AC component reflected from zone 4 and characterized by *R̃*_4_, being purely due to mechanical motion, is insensitive to any scattering losses as this wave is amplitude-modulated. Eqn (1) is then corrected by a prefactor of 0.28 that accounts for the 1.9 μm spot diameter of the probe Gaussian beam. See Fig. S3(a)† for more details on the scaling prefactor.

**Fig. 3:**
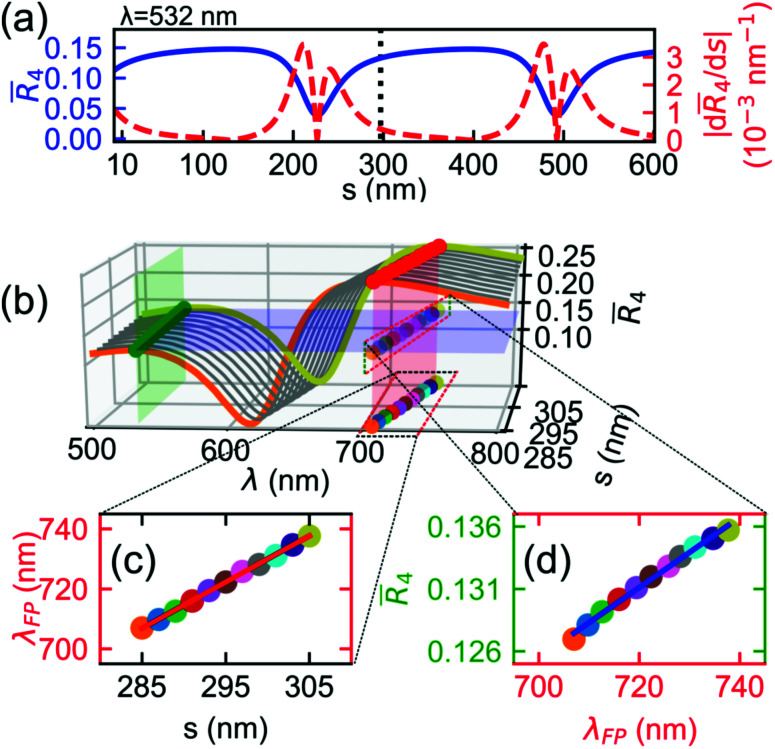
(a) Calculated reflectance *R̄*_4_ and device responsivity |d*R̄*_4_/d*s*| *vs.* vacuum spacer height *s* of the measured FP device evaluated at *h* = 55 nm and *λ* = 532 nm. (b) Waterfall plot of FP reflectance as a function of *λ* at varying *s*, with the used probe wavelength (green plane) situated at *λ* = 532 nm. (c) Colored scatter plot of *λ*_FP_ as a function of *s*. The slope of the red solid line originates from the intersection of the red plane with the *λ* − *s* plane in (b). (d) Colored scatter plot of *R̄*_4_ as a function of the cavity shift *λ*_FP_. The blue solid line originates from the intersection of the blue plane with the red plane in (b).

Estimation of |d*R̄*_4_/d*s*| requires the calculation of the gradient of the corrected *R̄*_4_ with respect to *s*. Fig. 3(a) shows *R̄*_4_ and its gradient as functions of *s*. As our NbSe_2_ plate is considered bulk,^27^ the *R̄*_4_*versus s* dependence shows a periodic yet non-sinusoidal behaviour. Yet, this dependence exhibits *λ*/2 periodicity, though the peak-to-dip and dip-to-peak spacings are asymmetric. The minima and maxima in the |d*R̄*_4_/d*s*| *versus s* response are shifted by about ±*λ*/12 with respect to the dip in *R̄*_4_*versus s*, deviating from the periodic *λ*/4 spacing expected for FP cavities with a partially transparent moving mirror. We also find that the *R̄*_4_*versus s* dependence for a monolayer NbSe_2_ flake follows a distorted, sinusoidal behaviour as shown in Fig. S4(a).† This is unlike the regular sinusoid shape for those mechanical resonators of optically thin cross-sections.^2,7^ Evaluating |d*R̄*_4_/d*s*| at *s* = *s*_drum_ (black dotted line) yields a device responsivity of 0.40 × 10^−3^ nm^−1^.

We define the average |d*R̄*_4_/d*s*| to account for spatial variations in *s*_drum_ across the plate due to the pressure difference and DC voltage. Note that each complex-valued refractive index is dependent on the probing wavelength; this translates to the wavelength-dependent *r*_h_, *r*_s_ and *Γ*_m_. We modeled |d*R̄*_4_/*s*|_avg._ by the chain rule |Δ*R̄*_4_(*λ*)/Δ*λ*_FP_‖Δ*λ*_FP_/Δ*s*|_*s* = *s*_drum__, where Δ*R̄*_4_/Δ*λ*_FP_ is the change of *R̄*_4_ with regards to the wavelength shift in the FP cavity, and Δ*λ*_FP_/Δ*s* is the wavelength shift of the FP cavity caused by the change of the spacer gap. Here, we define *λ*_FP_ as the wavelength at which *R̄*_4_(*λ*) is maximum in the range of *λ* = 500–900 nm. The resulting dependences are shown as a waterfall plot in Fig. 3(b) with a gap range exceeding the uncertainty of our stylus profilometer. Fig. 3(b) demonstrates larger *R̄*_4_ at near-infrared wavelengths. Fig. 3(c) shows the peak wavelength of the cavity, falling in the near-infrared range, shifting linearly with a slope of 1.543 nm/nm as *s*_drum_ increases from 285 nm to 305 nm. Fig. 3(d) shows how the shift consequently increases *R̄*_4_(*λ*) linearly, with a slope of 0.28 × 10^−6^ nm^−1^. The product of these two slopes, |d*R̄*_4_/d*s*|_avg_ = 0.43 × 10^−3^ nm^−1^, agrees with the evaluation in Fig. 3(a). The continuous behaviour seen in Fig. 3(b and c) is different from the discontinuous dependence observed for thin membranes in the same ranges of *s*, as shown in Fig. S4(b–d).† Their difference is mainly due to sharper features of the device responsivity of thin membranes as compared to thicker plates.

**Fig. 4:**
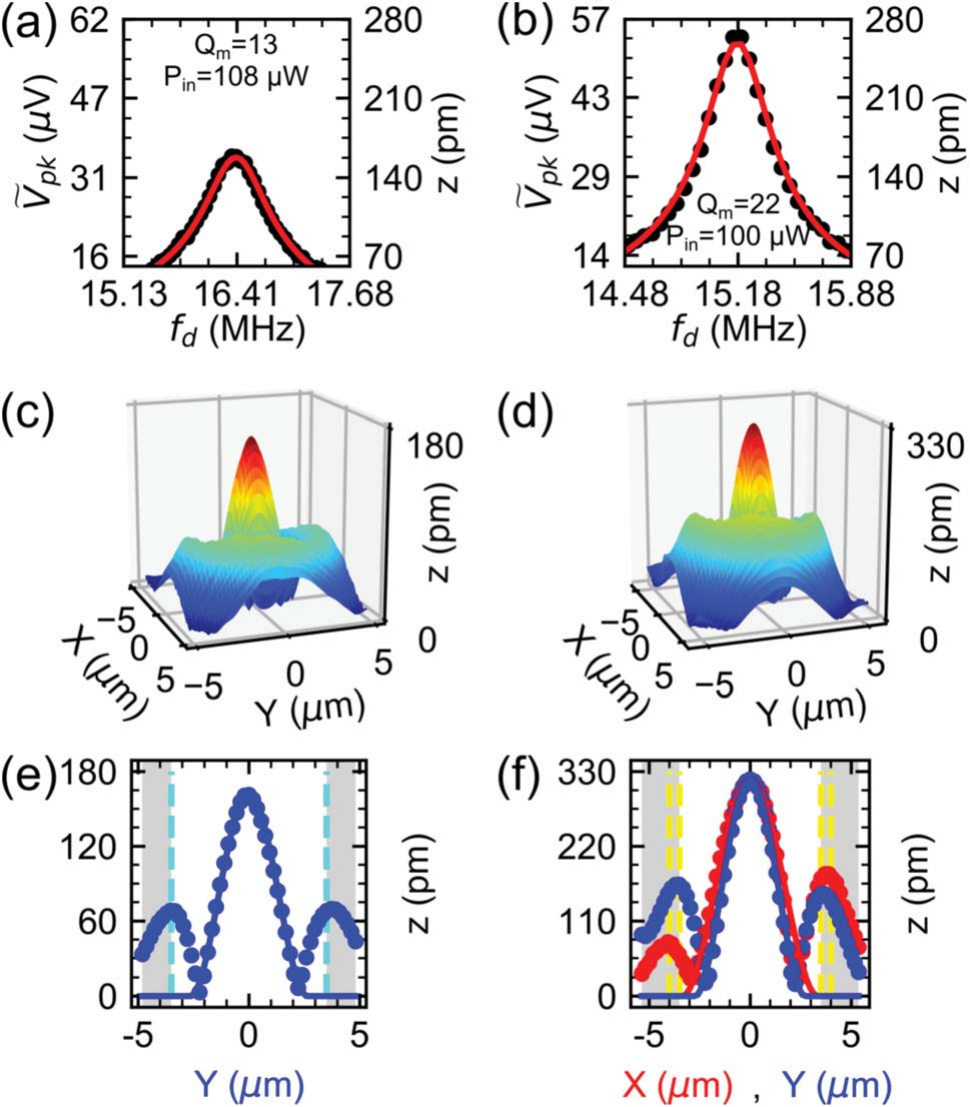
Spot-based displacement amplitude response (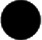) of device A (a) and device B (b) at *V*_DC_ = 4 V and *V*_AC_ = 125 mV and their driven resonator fits (red solid lines). The data shown in (c) and (d) refer to the spatial amplitude mode shape of devices A and B at *f*_d_ = *f*_m_. The amplitude profiles along *X* (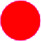) and *Y* (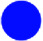) axes of the mode shapes for devices A (e) and B (f) are fitted with a clamped circular plate model (red and blue solid lines for *X* and *Y*, respectively). Dashed vertical lines indicate the edges of the holes.

We use the average responsivity together with the interferometer system gain *S*(*λ*) (V/W) (see the ESI† for more detailed calculations) and the laser probe power *P*_in_ to define the displacement amplitude *z* as2
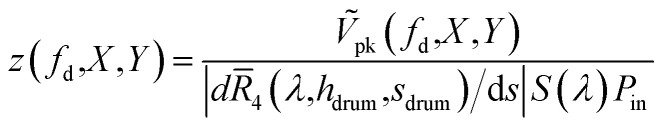
where *Ṽ*_pk_ is the frequency and position-dependent peak voltage response of the NMR. The denominator in eqn (2), when squared, represents the transduction factor *α* (in V^2^ m^−2^) that one can deduce from the measured Brownian motion of a mechanical resonator probed by an interferometric system.^18,19^ This quantity accounts for the device responsivity and the detection parameters in the interferometric setup. We deduce 
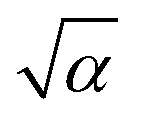
 factors of 0.20 μV pm^−1^ for device A and 0.22 μV pm^−1^ for device B for probe powers listed in Fig. 4(a and b).

### Calibration of motional amplitude in the frequency and spatial domains

Fig. 4(a and b) show the measured voltage response and its corresponding *z* for devices A and B. The measured *z* response profile agrees well with a driven mechanical resonator model in the linear regime:^42^3
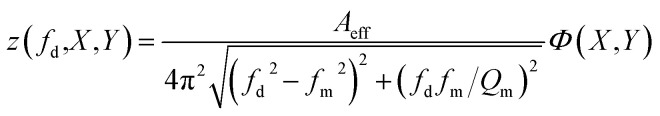
where *f*_m_ is the fundamental mode frequency, *Q*_m_ is the mode quality factor, and *A*_eff_ is the amplitude expressed as effective acceleration. *Φ*(*X*, *Y*) is the mode shape of the device described as4
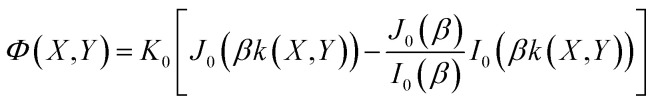
where *J*_0_ and *I*_0_ are the zeroth Bessel and modified Bessel functions of the first kind, respectively, *β* = 3.1961 is the fundamental root of the frequency equation for a clamped circular plate, and *K*_0_ = 0.947 is a normalization constant. Here, 
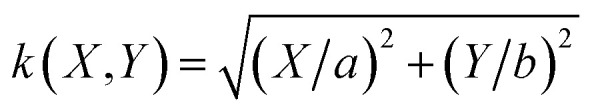
 represents the normalized coordinates away from the maximum of *z*, where *a* and *b* represent the NMR major (*X* axis) and minor (*Y* axis) modal radii, respectively. By setting *Φ*(*X*, *Y*) = 1, we measure *z*_A_ = 158 ± 2 pm for device A and *z*_B_ = 259 ± 3 pm for device B. Their magnitudes are three orders of magnitude smaller than *h*_drum_ and *s*_drum_.

By driving the plates at *f*_m_, and probing their spatial mode shape with scanning mirrors, we observe surface plots of *z* for devices A and B as shown in Fig. 4(c and d). Fig. 4(e and f) show *X* and *Y* axes cuts, with both axes intersecting at *z*_max_ of Fig. 4(c and d). They reveal *z* profiles that agree with eqn (4), with *a* and *b* acting as free parameters. *z*_max_, *a*, and *b* of the two plates are listed in Table 2. The discrepancy in the values of *z*_B_ and *z*_max_ of device B is due to the location of the laser spot that probed Fig. 4(b). While *z*_A_ lies at *X* = *Y* ≈ 0, *z*_B_ lies at *X*, *Y* ≈ 1 μm from the spatial peak. Both *a* and *b* for devices A and B are smaller than the hole radii (set as cyan and yellow dashed lines in Fig. 4(e and f)), making *f*_m_ for both devices higher than the designed values. A plausible explanation for this is that an initial slack is introduced in the system during the fabrication process.^43,44^ First, the flake is anchored to the spacer at the hole edge by van der Waals forces. Second, at room temperature, the annealed spacer, CSAR-62, which acts as a suspension support to the drumhead, is softer than typical NMR supports like SiO_2_^1,4,5^ and Au.^1,2,9^ These factors, which add up to clamping losses, contribute to the low *Q*_m_ measured for devices A and B. Furthermore, both devices suffer from imperfect, non-uniform clamping boundaries.^45^

**Table 2:** Modal properties of NbSe_2_ devices

Specifications	Device A	Device B	Method
*z*_max_ (pm)	161	320	Eqn (4)
*a* (μm)	2.7 ± 0.2	3.19 ± 0.06	Eqn (4)
*b* (μm)	2.6 ± 0.2	2.66 ± 0.02	Eqn (4)
*m*_eff_ (fg)	1.4	1.74	ESI
*A*_eff_ (km s^−2^)	132	132	Eqn (3)
*F*_eff_ (pN)	191	229	ESI
*E*_Y_ (GPa)	135 ± 13		ESI

Table 2 lists other NMR-related quantities that are derived from Fig. 4 such as the effective mass *m*_eff_, acceleration *A*_eff_, force *F*_eff_, and Young's elastic modulus *E*_Y_. These quantities are derived from a clamped elliptical plate model, with the details discussed in the following sections. The estimated *E*_Y_ is within the range of reported values^46,47^ for bulk NbSe_2_ flakes. These quantities are obtained without inducing damage on the flake, and are independent of the actuation scheme.

Eqn (4) does not explain the asymmetric sinusoidal waves propagating beyond the drum edges seen in Fig. 4(e and f). These waves are spatial signatures of support losses due to imperfect flake clamping at the edges.^48^ Discussing the waves' origin goes beyond the scope of this study, though resolving the waves' amplitude, which is 1/3 of *z*_max_, demonstrates the capability of our method to visualize acoustic waves in NMRs.^49,50^

### Merits of the method

Our motional amplitude calibration works as an *ab initio* technique because our model-based approach presupposes non-sinusoidal yet periodic Fabry–Perot reflectance *versus* spacer height which arises from the thickness-dependent optical properties of the suspended flake. With an extensive database that accounts for the effects of the number of layers on the complex-valued refractive indices of 2H-NbSe_2_, the proposed method can distinguish in great detail the thickness and the number of layers present in NbSe_2_ NMRs. The resolution in differentiating 1L, 2L, 3L, and multilayer 2H-NbSe_2_ is shown in Fig. S6(a).† Moreover, its corresponding *s* is traced as simulated in Fig. S6(b).† The high level of detail and contrast between the drum and clamps in the *h* and *s* profiles, as shown in Fig. 2(b and c), makes the confocal microscope an indispensable tool for the optical contrast scheme. Such microscopy, as compared to Raman spectroscopy, has been demonstrated previously to be faster and covers a larger mapping area in distinguishing the number of graphene layers supported by SiO_2_ (ref. 51) and SiC^52^ substrates.

The calibration method also complements the photodetector-based approach^12^ as the resonator does not need to be driven to a nonlinear regime of motion to trace its linear motion. Moreover, the values of 
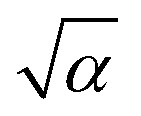
 acquired from our approach agree with those extracted from the measurement of Brownian motion from other studies as shown in Table 3. Also, see Fig. S7(a and b)† for the estimated power spectral density and Brownian motion of devices A and B. The simulated results confirm the validity of our method for both Brownian and linearly driven motion, which is sufficient for preliminary testing of integrated NMR devices.

**Table 3:** Application of the MIA calibration scheme to the work of other researchers

Device	*h* (nm)	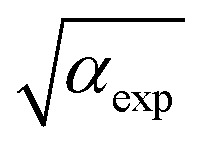 (μV pm^−1^)	*z*_exp_ (pm)	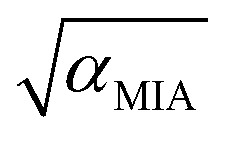 (μV pm^−1^)	*z*_MIA_ (pm)	Ref.
MoS_2_ plate	68.1	2.36	—	2.29	—	5
Graphene membrane	5	0.612	4000	0.614	4013	19
MoS_2_ membrane	0.665 (1L), 2.0 (3L)	0.100 (1L), 0.295 (3L)	—	0.102 (1L), 0.289 (3L)	—	7

Applying MIA for simulating optical-to-motional responsive 2H-NbSe_2_ resonators with the same substrate, probe laser wavelength, and vacuum gap, we interpret from Fig. S3(c and d)† that resonators with thicknesses below 30 nm have higher device responsivities than the experimental samples. For a vacuum gap of 85 nm, the smallest vacuum gap achieved for suspended van der Waals materials is conducive to microwave optomechanics^8,14^ and NMR-mediated cavity-qubit systems;^53^ these resonators, except for monolayer NbSe_2_, show higher device responsivity. 2H-NbSe_2_, apart from being a superconducting van der Waals material used for single-photon superconducting detectors,^54^ has the potential of being integrated with optoelectromechanical platforms.

## Conclusions

In summary, we have demonstrated an *in situ*, non-invasive method of calibrating motional displacement of NbSe_2_ NMRs by exploiting wave interference phenomena in FP cavities. Using a probe laser beam, and applying MIA, we have first determined the cross-sectional profiles of the NMR thickness and spacer height through contrast minimization. Then the transduction factors of NbSe_2_ plate resonators are extracted to convert the measured signal to the actual motional amplitudes, which turned out to be in the order of hundreds of picometers. This information provides access to the fundamental mode properties of the drumheads. These include the femtogram effective mass of the drumheads, the 135 ± 13 GPa Young's modulus of the drum material, and the hundreds of piconewton driving force felt by the drumheads.

We note that this method has been instrumental in investigating the superposition of vibrational modes in plate NMRs induced by off-resonant frequency driving.^30^ We foresee that this method, not limited to van der Waals materials, can be extended to flexural NMRs and acoustic wave resonators. Lastly, this work may help establish FP laser interferometry as a non-invasive tool for evaluating NMR specifications that are integrated into other solid-state components like superconducting qubits, photonic cavities, and NMR arrays.

## Author contributions

C.-D. C. conceived the devices and supervised the project. J. C. E. fabricated the devices. M. A. C. A. and J.-Y. W. modeled the calibration scheme. C.-Y. Y. and K.-H. L. designed and built the setup for optical measurements. M. A. C. A., J. C. E. and C.-Y. Y. performed the experiment. M. A. C. A., J. C. E., J.-Y. W., T.-H. L., K.-S. C.-L., S. K., Y. P. and C.-D. C. analyzed the data, performed simulations and wrote the manuscript; all authors discussed the results and contributed to the manuscript.

## Conflicts of interest

There are no conflicts to declare.

## Supplementary Material

NA-004-D1NA00794G-s001
