# Supplementary material for: Fabry–Perot interferometric calibration of van der Waals material-based nanomechanical resonators
Source: Nanoscale Adv. 2021 Nov 23;4(2):502–9. doi: 10.1039/d1na00794g (PMC9416946; doi:10.1039/d1na00794g)
Supplement: NA-004-D1NA00794G-s001 [file NA-004-D1NA00794G-s001.pdf]

Electronic Supplementary Material (ESI) for Nanoscale Advances.
This journal is © The Royal Society of Chemistry 2021

Electronic Supplementary Information: Fabry-Perot Interferometric Calibration of van der Waals Material-Based Nanomechanical Resonators

Myrron Albert C. Aguila,[*,†,‡,¶] Joshoua C. Esmenda,[*,†,‡,¶] Jyh-Yang Wang,[¶] Teik-Hui Lee,[¶] Kung-Hsuan Lin,[¶] Chi-Yuan Yang,[¶] Kuei-Shu Chang-Liao,[†] Sergey Kafanov,[§] Yuri Pashkin,[§] and Chii Dong Chen[*,¶]

†*Department of Engineering and System Science, National Tsing Hua University, Hsinchu, Taiwan*

‡*Nano Science and Technology Program, Taiwan International Graduate Program, Academia Sinica and National Tsing Hua University*

¶*Institute of Physics, Academia Sinica, Taipei, Taiwan*

§*Department of Physics, Lancaster University Lancaster LA1 4YB, United Kingdom*

E-mail: maguila@gate.sinica.edu.tw; jesmenda@gate.sinica.edu.tw; chiidong@phys.sinica.edu.tw

Phone: +886 (2)2785 8955. Fax: +886 (2)2785 4187

Additional Details about Experimental Methods

Details about Interferometric Detection Scheme

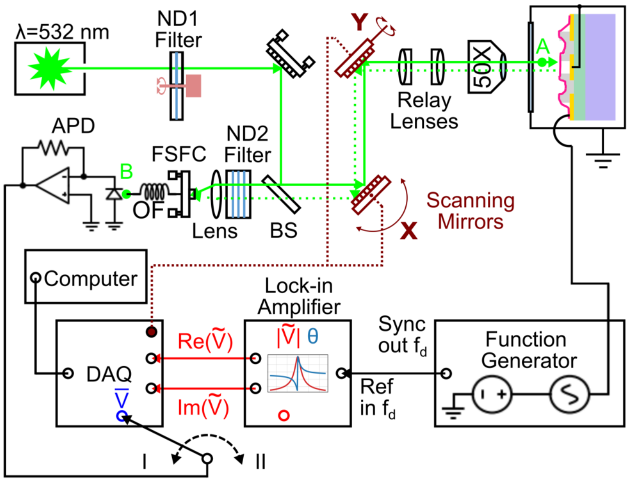

Figure S1: Experimental setup for electromotive actuation and interferometric calibration of $NbSe_2$ drum plate resonators.

The drums are actuated with a function generator, with its excitation frequency f_d made as reference for the lock-in amplifier. The incident laser beam (green solid line at green point labeled "A") has its input power P_{in} tuned with a variable neutral density (ND1) filter, and attenuated with a 50:50 non-polarizing beam splitter (BS), and other mirrors. The location of the laser spot is controlled with two scanning mirrors, one tilting in the X direction and the other tilting in the Y direction. The laser beam diameter is then expanded in the relay lenses before it is focused on the drum through the 50X microscope objective. The reflected light (green dotted line) passes through the BS, and the convex lens. The focused light is coupled by the free space fiber coupler (FSFC), and is transmitted through a multimode optical fiber (OF). Finally, the avalanche photodetector (APD) receives the incident power of light from point B (green point labeled "B"). To prevent the received light from reaching the saturation power of the APD, an ND filter (ND2) with 1% transmission is placed between the lens and the BS. All of the optical components are used to capture the confocal image, and visualize the mode shape of the drums.

Atomic Force Microscopy Measurements

To verify the accuracy of the proposed profilometric approach using optical contrast, we perform atomic force microscopy measurements under tapping mode using the Veeco Di-Innova Atomic Force Microscope. Figure S2(a-b) shows AFM topographical scans of the $NbSe_2$ edges nearest to the drumhead location. The thickness profiles, shown in Fig. S2(c-d), show that the thickness extracted near the drums ranges from 53.4 - 57.3 nm. We obtain $h = 55.4 \pm 0.6\,\text{nm}$ from averaging the thickness measured from the two scans, which agrees well with the results obtained by optical contrast.

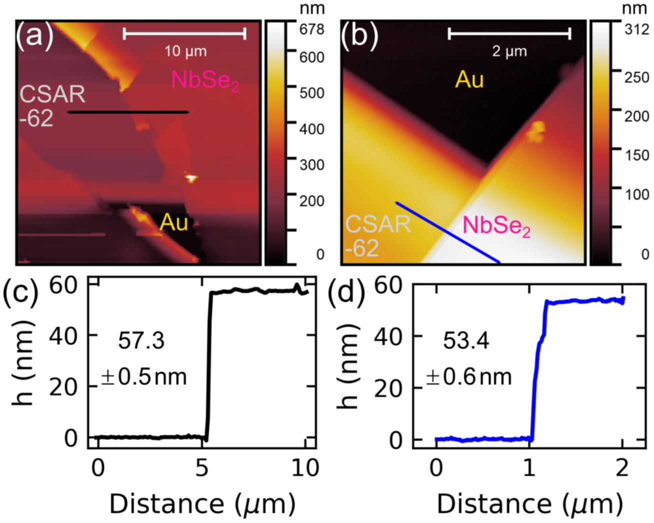

Figure S2: (a-b) AFM topographical images of edges of the large-area $NbSe_2$ flake that is supported by the CSAR-62 electron-beam resist. (c) h profile of $NbSe_2$ thin film as captured from the black solid line cut in (a). (d) Height profile of $NbSe_2$ thin film as captured from the blue line profile in (b).

Generalized Reflection Amplitude Recursion Method for Calculating Overall Reflectivity

In this section, we use the convention set in Table 1 in building the modelled reflectance Eq. (1) in the main manuscript. We make use of the Multilayer Interference Approach (MIA)[1,2] to estimate the total reflectance $\overline{R}_1$ of the $NbSe_2$ multilayer stack with $N - 1$

Table 1: Notation between the Main Manuscript and the Supporting Information (SI). Indices in the main manuscript refer to zones whereas indices in SI refer to the layer numbers in the recursion.

Main Text	Supporting Information for derivation
r_h	r_1
r_s	r_2
δ_h	δ_1
δ_s	δ_2
Γ_m	Γ_3
$\overline{R}_1$	$\overline{R}_3$
$\overline{R}_2$	$\overline{R}_2$
$\overline{R}_3$	$\overline{R}_1$ (Spacer=CSAR-62)
$\overline{R}_4$	$\overline{R}_1$ (Spacer=vacuum)

interfaces and N layers as described in the main manuscript. Each material layer with index i ($i = 0, 1, ..., N-1$) has a complex-valued refractive index $\hat{n}_i = \hat{n}_i(\lambda) = \mathrm{Re}\,(\hat{n}_i) - j\mathrm{Im}\,(\hat{n}_i)$, and thickness h_i. In general, light hits the layer at an incident angle θ_i. Most materials in the multilayer stack, except CSAR-62 and SiO_2, absorbs light, implying $\mathrm{Im}\,(\hat{n}_i) > 0$. We assume that coherent light with wavelength λ originates from a point source within vacuum. Light then penetrates the layer with its speed retarded by $\mathrm{Re}\,(\hat{n}_i)$, and its intensity attenuated by $\mathrm{Im}\,(\hat{n}_i)$. The ith layer then possesses an optical phase thickness

$$\delta_i = \frac{2\pi\hat{n}_i}{\lambda} h_i \cos\theta_i \tag{S1}$$

that represents propagation of coherent light in a thin-film layer. MIA assumes that the initial and final layers have semi-infinite thickness ($h_0 = h_{N-1} \gg 10\lambda$).[3]

The recursion starts from the bottom interface $i = N-2$, which is located between a finite thin film of layer $i = N-2$ and the substrate of layer $i = N-1$. The bare reflection coefficient of the bottom layer is then reduced to $\Gamma_{N-1} = r_{N-1}$, where r_{N-1} is the Fresnel coefficient of the $i = N-2$ interface. The Fresnel coefficient of the ith interface is expressed

as

$$r_i^{TE} = \frac{\hat{n}_{i-1}\cos\theta_{i-1} - \hat{n}_i\cos\theta_i}{\hat{n}_{i-1}\cos\theta_{i-1} + \hat{n}_i\cos\theta_i} \tag{S2a}$$

$$r_i^{TM} = \frac{\hat{n}_{i-1}/\cos\theta_{i-1} - \hat{n}_i/\cos\theta_i}{\hat{n}_{i-1}/\cos\theta_{i-1} + \hat{n}_i/\cos\theta_i} \tag{S2b}$$

where the superscripts TE and TM refer to transverse electric (s-) polarization, and transverse magnetic (p-) polarization, respectively. The reflection coefficient of the upper interface Γ_i is evaluated as

$$\Gamma_i = \frac{r_i + \Gamma_{i+1}e^{-2j\delta_i}}{1 + r_i\Gamma_{i+1}e^{-2j\delta_i}} \tag{S3}$$

The goal of the recursion is to evaluate Γ_i using the previously evaluated reflection coefficient Γ_{i+1} until the reflection coefficient that contains the refractive index of the top layer is evaluated. We then multiply this coefficient by its complex conjugate to obtain the point-source reflectivity of the multilayer stack. The polarization angle of light that the probe laser emits is close to 45°, resembling unpolarized light. We then calculate the overall stationary reflectance as

$$\overline{R}_i = \Gamma_i^*\Gamma_i = |\Gamma_i|^2 \tag{S4}$$

For unpolarized light, Eq. (S4) is calculated for both TE and TM polarizations, and then averaged.

In determining the optical contrast, the Gaussian intensity distribution of the probe beam can be ignored.[4] The Fresnel coefficients Eq. (S2) in each layer in the multilayer stack are then evaluated at normal incidence as

$$r_i(\theta_{i-1} = 0, \theta_i = 0) = \frac{\hat{n}_{i-1} - \hat{n}_i}{\hat{n}_{i-1} + \hat{n}_i} \tag{S5}$$

and Eq. (S1) is reduced to $\delta_i = 2\pi\hat{n}_i h_i/\lambda$. We evaluate the reflection coefficient of the mirror

Γ_3 by evaluating Eq. (S3) from interface $i = 6$ to $i = 3$.

$$\Gamma_3 = \frac{r_3 + r_4 e^{-2j\delta_3} + r_4 r_5 e^{-2jA} + r_6 e^{-2jD} + G r_5 e^{-2j\delta_4} + r_3 H e^{-2j\delta_5} + G r_6 e^{-2jB} + H e^{-2jC}}{1 + G e^{-2j\delta_3} + r_3 r_5 e^{-2jA} + r_3 r_6 e^{-2jD} + r_4 r_5 e^{-2j\delta_4} + H e^{-2j\delta_5} + r_4 r_6 e^{-2jB} + G H e^{-2jC}} \tag{S6}$$

where

$$\begin{aligned} &A = \delta_3 + \delta_4; B = \delta_4 + \delta_5; \\ &C = \delta_3 + \delta_5; D = \delta_3 + \delta_4 + \delta_5; \\ &G = r_3 r_4; H = r_5 r_6 \end{aligned} \tag{S7}$$

In this expression, r_3, r_4, r_5 and r_6 denote the reflection coefficient of the spacer-Au, Au-Cr, Cr-SiO_2 and SiO_2-Si interfaces, respectively. The reflection coefficient of the bare stationary mirror is $r_3 = (1 - \hat{n}_3)/(1 + \hat{n}_3)$ and its corresponding reflectance is $\overline{R}_3 = \Gamma_3^* \Gamma_3$. We note that the Au-Cr layer contributes largely to the value of $\overline{R}_3$.

The recursive reflection coefficient for the CSAR-62 covered mirror can be written as

$$\Gamma_2^{'} = \frac{r_2^{'} + \Gamma_3 e^{-2j\delta_2}}{1 + r_2^{'} \Gamma_3 e^{-2j\delta_2}} \tag{S8}$$

where δ_2 is the optical phase thickness of the spacer, and $r_2^{'} = (1 - \hat{n}_2)/(1 + \hat{n}_2)$ represents the reflection coefficient of the top interface of the spacer. The reflectance of the CSAR-62 covered stationary mirror is $\overline{R}_2 = \Gamma_2^{'*} \Gamma_2^{'}$.

The reflection recursion coefficient of the spacer-covered Fabry-Perot cavity can be written as

$$\Gamma_1 = \frac{r_1 + r_2 e^{-2j\delta_1} + \left[r_1 r_2 + e^{-2j\delta_1}\right] \Gamma_3 e^{-2j\delta_2}}{1 + r_1 r_2 e^{-2j\delta_1} + \left[r_2 + r_1 e^{-2j\delta_1}\right] \Gamma_3 e^{-2j\delta_2}} \tag{S9}$$

where δ_1 is the optical phase thickness of the $NbSe_2$ flake. Multiplying Eq. (S9) with its complex conjugate results in the FP reflectance $\overline{R}_1 = \Gamma_1^* \Gamma_1$ as indicated in Eq. (1) in the main manuscript. If the spacer is vacuum, $r_2 = -r_1$, and the total reflectance of the main

FP cavity with a movable mirror is

$$\overline{R}_1 = \left| \frac{r_1 \left(1 - e^{-2j\delta_1}\right) - \left(r_1^2 - e^{-2j\delta_1}\right) \Gamma_3 e^{-2j\delta_2}}{1 - r_1^2 e^{-2j\delta_1} - r_1 \left(1 - e^{-2j\delta_1}\right) \Gamma_3 e^{-2j\delta_2}} \right|^2 \tag{S10}$$

Table 2: Refractive index database of movable and stationary Bulk $NbSe_2$ FP cavities at $\lambda = 532\,\mathrm{nm}$.

i	Material	$\hat{n}_i$	h_i (nm)	$r_i\,(\theta_i = 0)$	$\delta_i(2\pi)$
0	Vacuum	1	∞	-	∞
1	Bulk $NbSe_2$[5]	3.07 - 1.00j	55.14	-0.54 + 0.11j	0.32 - 0.10j
2	Vacuum	1	297.2	0.54 - 0.11j	0.56
	CSAR-62[a]	1.5087	296.0	0.37 - 0.14j	0.85
3	Au[6]	0.48 - 2.36j	40	-0.62 + 0.61j[b]	0.04 - 0.18j
				-0.37 + 0.75j[c]	
4	Cr[6]	3.04 - 3.33j	20	-0.33 - 0.25j	0.11 - 0.13j
5	SiO_2[6]	1.46	543	0.58 - 0.31j	1.49
6	Si[6]	4.15 - 0.04j	∞	-0.48 + 0.004j	∞

[a] 2% smaller than specified.[7]

[b] vacuum-Au interface. [c] CSAR-62 - Au interface.

To mimic our experimental setup, we also consider the setup's effective numerical aperture (NA). The NA sets the spot size of the beam, and thereby the resolution of the system. The reflectivity of the surface probed by a Gaussian beam source can be written as

$$\overline{R}_{1G} = \int_0^{\theta_{NA}} \left|\Gamma_1(\theta)\right|^2 \exp\left(-2\frac{\sin^2\theta}{\sin^2\theta_{NA}}\right) d\theta \tag{S11}$$

where $\theta_{NA} = \sin^{-1}(NA)$ is the maximum collection angle of the interferometric setup. Eq. (S11) represents the weighted average intensities of light reflected from different angle of incidence for a finite spot size. Using relevant quantities listed in Table 2, the effect of NA on both the resulting $\overline{R}_1$ and $\left|d\overline{R}_1/ds\right|$ is simulated, and then best fitted with a power-law scaling factor γ, as shown in Fig. S3. For convenience, we approximate Eq. (S11) with a corrected reflectance

$$\overline{R}_{1G} \approx \gamma \overline{R}_1 \tag{S12}$$

where $\gamma = 1.78(NA)^{1.78}$. For an estimated spot size of 1.9 μm, $NA \approx 0.35$ and $\gamma = 0.28$. γ is used to correct the values of the $\overline{R}_4$ and $\left|d\overline{R}_4/ds\right|$ in both the main manuscript, and the following sections.

Effect of Thickness of $NbSe_2$ resonators on its Reflectance and Device Responsivity Profile

Table 3: Refractive Index and Fresnel coefficient of 1L, 2L and 3L $NbSe_2$ at $\lambda = 532$ nm based on the measured dielectric constants provided by Hill and coworkers[5]

Number of Layers	$\hat{n}_{\text{drum}}$	$r_h\,(\theta_h = 0)$
1L	$6.48 - 1.69j$	-0.746 + 0.058j
2L	$5.42 - 1.24j$	-0.700 + 0.058j
3L	$4.96 - 1.18j$	-0.677 + 0.064j

We apply the method of Wang and Feng[8] to convert the complex dielectric constants of monolayer (1L), bilayer (2L), trilayer (3L) and bulk pristine $NbSe_2$ at $\lambda = 532$ nm to complex-valued refractive indices, which are listed in Tables 2-3. Both the real and imaginary parts of the refractive indices of $NbSe_2$ at $\lambda = 532$ nm decrease when the number of layers increases, showing the thickness effects on the optical properties of $NbSe_2$ flakes. We assume that the refractive index of $NbSe_2$ having ten layers is equal to that of the bulk. Based on the work of Darvishzadeh and coworkers[9] in estimating the change in the refractive index from in-plane strain, the estimated motional strain values, 3×10^{-5} for device A and 6×10^{-5} for device B in comparison to the measured minor axis diameter, give negligible refractive index change roughly in the order of 10^{-4}.

Estimation of the optical-to-motional device responsivity $\left|d\overline{R}_4/ds\right|$ requires the calculation of the gradient of the corrected $\overline{R}_4$ with respect to a vacuum gap of height s. Figures S3(b-c) show $\overline{R}_4$ and its gradient as functions of s. As our $NbSe_2$ plate is considered bulk,[5] the $\overline{R}_4$ versus s dependence shows a periodic yet non-sinusoidal behaviour. Yet, this dependence exhibits $\lambda/2$ periodicity, though the peak-to-dip and dip-to-peak spacings are

asymmetric. $\left|d\overline{R}_4/ds\right|$ depends on the steepness of the $\overline{R}_4\,(s)$ dependence. The minima and maxima in the $\left|d\overline{R}_4/ds\right|$ versus s response are shifted by about $\pm\lambda/12$ with respect to the dip in $\overline{R}_4$ versus s, deviating from the periodic $\lambda/4$ spacing expected for conventional FP cavities. Evaluation of $\left|d\overline{R}_4/ds\right|$ at $s = s_{drum}$ (vertical black dotted line) gives the device responsivity of $0.40 \times 10^{-3}\,\mathrm{nm}^{-1}$. As the thickness h of the plate resonator decreases, we see a qualitative change in the $\overline{R}_4\,(s)$ dependence as shown in Fig. S3(b). The periodic $\overline{R}_4\,(s)$ lineshape transforms from an inverted hanger lineshape at a "bulk" thickness $h = 38.4 - 60\,\mathrm{nm}$ (64-100 layers), to a Fano-resonance lineshape at an "intermediate" thickness $h = 12 - 37.8\,\mathrm{nm}$ (20-63 layers), and lastly to a distorted sine wave at a "multilayer" thickness of $h = 6 - 11.4\,\mathrm{nm}$ (10-19 layers). During the transformation, $\lambda/2$ periodicity is preserved. Despite the dependence of the refractive index of $NbSe_2$ flakes on the number of layers when the number is small as listed in Table 3, we see that the $\overline{R}_4\,(s)$ dependence of monolayer, bilayer, and trilayer $NbSe_2$ flakes resembles that of the same material having ten layers, though the $\overline{R}_4$ range increases with increasing the layer thickness up to about $h = 30\,\mathrm{nm}$ (50 layers). When the number of layers exceed 50, the optical absorption of the flake, represented by a large $\mathrm{Im}(\delta_h)$, becomes significant and this reduces the slope of the $\overline{R}_4\,(s)$ dependence by $\sim e^{-2\mathrm{Im}(\delta_h)}$. On the contrary, when the number of layers is less than 50, the increased absorption results in a steeper $\overline{R}_4(s)$ dependence.

Figures S3(c-d) shows the effect of decreasing h in the $\left|d\overline{R}_4/ds\right|(s)$ dependence. Both plots reveal, besides the main peak '1', the appearance and disappearance of extra peaks '2' and '3' in the $\left|d\overline{R}_4/ds\right|(s)$ dependence in a certain range of thicknesses as traced in Fig. S3(d). Because the whole dependence is periodic, we inspect the local maxima of the $\left|d\overline{R}_4/ds\right|(s)$ dependence in the range $s = 100 - 400\,\mathrm{nm}$, which roughly corresponds to one period.

The values of $\left|d\overline{R}_4/ds\right|(s)$ is governed by two mechanisms:[10] interference and modulated absorption. Interference occurs when fraction of the light reflected from the bottom stationary mirror superpose with fractions of light reflected from the top and bottom interface

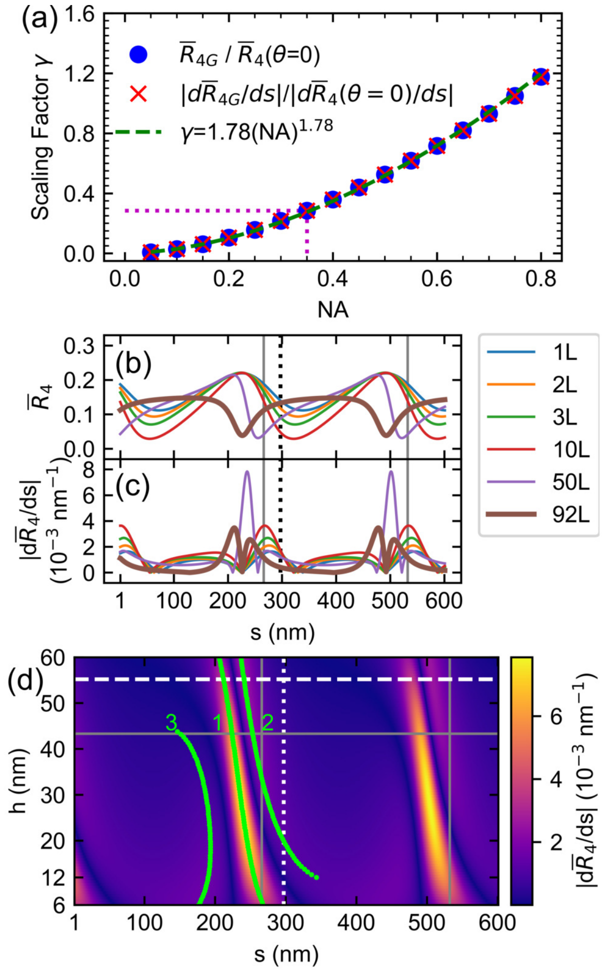

Figure S3: (a) Graph showing the dependence of the empirical scaling factor γ versus numerical aperture NA. The scaling factor agrees with the amplitudes of the Gaussian-distributed reflectance $\overline{R}_4$ and responsivity $\left|d\overline{R}_4/ds\right|$ normalized to their point source amplitudes. The curves are plotted at h=55.2 nm and s=297 nm. Purple dotted lines point to NA= 0.35. (b) $\overline{R}_4$ vs s profiles of $NbSe_2$ drum plates at varying plate thickness h. Each layer L has 0.6 nm in thickness. (c) device responsivity $\left|d\overline{R}_4/ds\right|$ vs s of $NbSe_2$ drum plates as a function of h. (d) Color map of the FP device responsivity as a function of h and s. For bulk $NbSe_2$ (10L, 6 nm), we plot the responsivity of bulk $NbSe_2$ (10-100L) at varying h. Dashed and dotted white lines correspond to the parameters measured from our devices. Lime lines corresponds to responsivity peaks located at certain values of h and s. Horizontal solid gray line in (d) corresponds to $h = \lambda/(4 \times \mathrm{Re}\,(\hat{n}_h))$. Vertical solid gray lines shown in (b-d) correspond to constructive interference of an ideal FP cavity with a vacuum gap, $s = m\lambda/2$, where m is an integer. All plots were simulated with $\lambda = 532$ nm.

of the movable plate. Interference is a result of the differences in the total optical phase thickness, which is affected largely with δ_s. Modulated absorption occurs when a standing wave is created between the stationary mirror and the movable plate, as a result of zero electric field condition in the metal-vacuum interface. This mechanism is influenced with both h and s.

The main peak, labelled '1' on the curved lime line in Fig. S3(d), originates from both interference effects and modulated absorption. Peak '1' shifts nonlinearly at decreasing thicknesses, with the vacuum gap of peak '1' s_1 approaching $\lambda/2$. Within the range of "bulk" thickness, modulate absorption dominate. Less intensity of amplitude-modulated light is reflected back to the detector due to multiple photon round trips and absorption losses within the vacuum gap of the FP cavity. Within the range of "multilayer" thickness, interference effects dominate. The reflected amplitude-modulated light travels at least one round trip within the vacuum gap before it is received by the photodetector. Within the range of intermediate thicknesses, both modulated absorption and interference effects contribute largely to the behaviour in s_1.

Peak '2', found at a vacuum gap of height s_2 and at thicknesses ranging from "bulk" to "intermediate", shifts nonlinearly from $s_2 = 237\,\text{nm}$ to $s_2 = 346\,\text{nm}$ at decreasing thickness before it vanishes completely at $h = 11.4\,\text{nm}$. Peak '3' is found at a vacuum gap of height $s_3 = 147\,\text{nm}$ and $h = \lambda/(4 \times \text{Re}\,(\hat{n}_h)) = 43.2\,\text{nm}$, the upper limit of "intermediate" thickness. It then shifts nonlinearly at decreasing h and persists up to $s_3 = 177\,\text{nm}$ for $h = 6\,\text{nm}$.

Next, we focus on Fig. S4(a) for the reflectance and responsivity of a monolayer $NbSe_2$ device as a function of s. The $\overline{R}_4\,(s)$ dependence resembles that of a distorted sine wave. The maxima in the $\overline{R}_4\,(s)$ dependence are located at $s = (m\lambda/2) - 40\,\text{nm}$. Moreover, each maximum in the $\overline{R}_4\,(s)$ dependence has distinct spacing from its neighboring minima. Within the range of $s = 100 - 400\,\text{nm}$ in the same plot, the $\left|d\overline{R}_4/ds\right|$ peak '1' appears at $s_3 = 280\,\text{nm}$ while the $\left|d\overline{R}_4/ds\right|$ peak '3' appears at $s_1 = 156\,\text{nm}$. A black dotted line at $s = 297\,\text{nm}$ points to a device responsivity of $1.390 \times 10^{-3}/\text{nm}$, highlighting that our current substrate is ideal

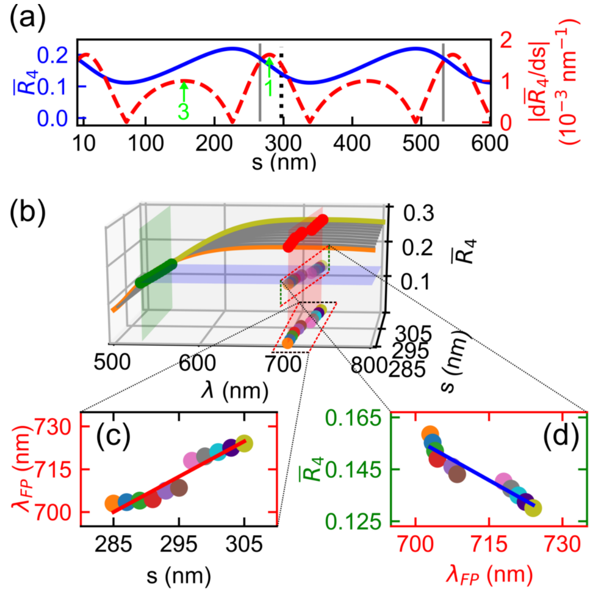

Figure S4: *Average device responsivity of a monolayer* $NbSe_2$ *FP cavity.* (a) Calculated reflectance $\overline{R}_4$ and device responsivity $\left|d\overline{R}_4/ds\right|$ vs vacuum spacer height s of the measured FP device evaluated at $h = 0.6$ nm and $\lambda = 532$ nm. A dotted black line refers to $s_{drum} = 297$ nm. Vertical solid gray lines correspond to $s = m\lambda/2$ periodicity, where m is an integer. (b) Waterfall plot of FP reflectivity as a function of λ at varying s, with our probe wavelength (green plane) situated at $\lambda = 532$ nm. (c) Colored scatter plot of the peak cavity wavelength λ_{FP} as a function of s. The slope of the red solid line originates from the intersection of the red plane with the $\lambda - s$ plane in (b). (d) Colored scatter plot of $\overline{R}_4$ as a function of λ_{FP}. The blue solid line comes from the the intersection of the blue plane with the red plane in (b).

for FP motional detection for the monolayer $NbSe_2$ case.

We modeled $\left|d\overline{R}_4/s\right|_{avg}$ for a monolayer $NbSe_2$ optical membrane using the chain rule cited in the main manuscript to demonstrate the reproducibility of the method. $\left|d\overline{R}_4/s\right|_{avg}$ is obtained from analyzing the waterfall plot of $\overline{R}_4$ versus λ dependence within the range of $s = 285 - 305\,\text{nm}$, as shown in Fig. S4(b). Figure S4(b) shows larger $\overline{R}_4$ range than Fig. S4(a), with the flake reflecting more at near-infrared wavelengths. The $\overline{R}_4$ versus λ dependence is totally different from Fig. 3(a) in the main manuscript, implying that the thickness of the flake affect the reflectance. Figure S4(c) shows the wavelength of the cavity with the maximum $\overline{R}_4$, falling in the near-infrared range, shifting parabolically as s increases from 285 nm to 305 nm. Nevertheless, the linear fit is used to extract the average slope of 1.239 nm/nm. Figure S4(d) shows how the shift consequently decreases $\overline{R}_4\,(\lambda)$ nonlinearly. The linear fit gives an average slope of -1.052×10^{-3}/nm. The product of these two slopes, $\left|d\overline{R}_4/ds\right|_{avg} = 1.30 \times 10^{-3}$/nm, agrees with $\left|d\overline{R}_4/ds\right|$ evaluated in Fig. S4(a). This quantity is about three times greater than what was reported in the main manuscript for a thicker plate.

Contrast Extraction Algorithm

Converting the voltages along the dashed lines in Figure 1(c) of the main manuscript to contrast values results in contrast profiles for devices A and B, as shown in Figs. S5(a-b), with devices A and B darker than its mirror references. Furthermore, the clamps are darker than the drums, with recorded contrast differences of around 0.07 for device A and 0.10 for device B. The change in the dependence of C_{exp} versus X can be attributed with the sudden change in the refractive index. The Limited-Memory Broyden-Fletcher-Goldfarb-Shanno (L-BFGS) algorithm[11] is used to minimize the difference between the experimental and modelled optical contrast, and determine h and s. We see that such algorithm assumes a linear relation of h and s with regards to a contrast value as shown from the intensity plots

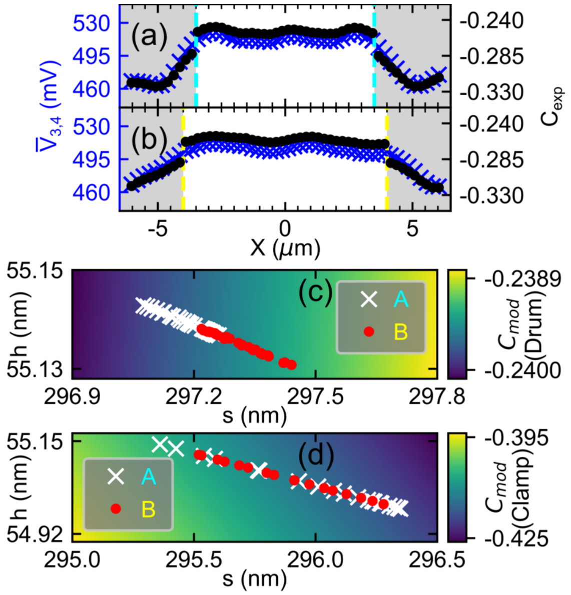

Figure S5: Additional details regarding the profilometric measurement of $NbSe_2$ drum plates via optical Rayleigh contrast. Raw data of the measured voltage and its corresponding contrast of (a) circular and (b) elliptical drum plates. The resulting X-axis cross-sections of both devices, based on the data acquired from Figure 3(c-d) of the main manuscript, are shown in (c) and (d).

Fig. S5(c-d) for both drum and clamp zones.

Using the contrast extraction implementation of MIA makes differentiating suspended monolayer (1L), bilayer (2L), trilayer (3L), and bulk $NbSe_2$ flakes possible. Figure S5(a) shows the distinction between 1L, 2L, 3L, and bulk $NbSe_2$ (assumed to be 10L and above) of increasing drumhead thickness h at a vacuum spacer height $s = 297\,nm$. The missing points from 4L to 9L imply the absence of information regarding the complex-valued refractive index of $NbSe_2$ at $\lambda = 532\,nm$. Figure S6(b) shows the traceability of s from C_{mod} at a certain number of layers. C_{mod} for few-layer (1-3L) $NbSe_2$ NMRs are shown to decrease nonlinearly at increasing gap height, with the 3L NMR demonstrating a steeper downward slope. $NbSe_2$ NMRs with intermediate thicknesses (50L and 92L) show a linear increase in C_{mod} at increasing s, with the thicker NMR demonstrating a gradual incline. The reversal in the C_{mod} versus s trend between 3L and 50L suggests a thickness regime where C_{mod} is constant at increasing s.

The main contributor to the noise floor, or best described as standard error, in our h and

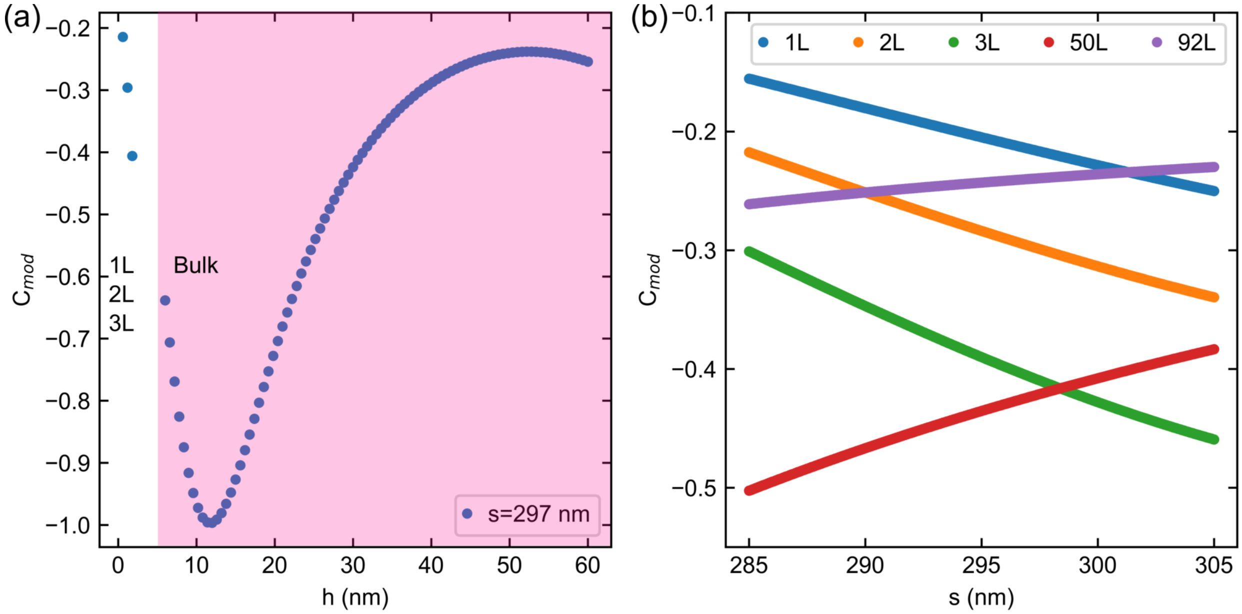

Figure S6: Differentiating C_{mod} between different h and s of $NbSe_2$ based on number of layers. (a) Modelled Contrast for different number of layers of $NbSe_2$. (b) C_{mod} as a function of s at different number of layers. The average intensity in zone 1 is used as reference in the calculation.

s_{drum} estimation would come from the variations in the surface morphology of the references, zones 1 and 2, that the interferometric setup has resolved in the confocal image shown in Fig. 2(a) in the manuscript. Acquiring C_{exp} entails determining the average intensity from the surface of zone 1 ($\overline{V}_1$=864 mV), and zone 2 ($\overline{V}_2$=906 mV). The h and s profiles in Figures 2(b-c) ignores the influence of the standard deviations of zone 1 ($\delta\overline{V}_1$=29 mV) and zone ($\delta\overline{V}_2$=64 mV) for each pixels of the drum and clamp zones. Accounting for the propagation of errors brought upon by the references would mean that the contrast errors are dependent on factors such as the smoothness of the reference zones, which would be non-trivially related to the resolution of the FP interferometer setup. For a simple estimate of the average error observed in the experiment, assuming that $\delta\overline{R}_{1,2} = \delta\overline{V}_{1,2}/\overline{V}_{1,2}$, we express the contrast error as

$$\Delta C_{mod} = C_{mod}\delta\overline{R}_{1,2}\left(\frac{1}{\overline{R}_{3,4} - \overline{R}_{1,2}} + \frac{1}{\overline{R}_{3,4} + \overline{R}_{1,2}}\right) \tag{S13}$$

where C_{mod} is the modelled contrast between the suspended flake and the bare mirror, as

shown in Figure S6. For suspended samples, the expected ΔC_{mod} for 1L, 2L, and 3L, $NbSe_2$ are 20×10^{-3}, 19×10^{-3}, and 17×10^{-3} respectively. These contrast errors are much smaller than the observed C_{mod} jumps from 1-3L, proving that the contrast method can distinguish few-layers with ease.

Estimation of Mass, Force and Young's Elastic Modulus of Circular and Elliptical $NbSe_2$ Drums

The effective mass of a clamped elliptical drumhead resonating at its fundamental mode is estimated as

$$m_{eff} = \xi \pi \rho a b h_{drum} \tag{S14}$$

where $\xi = 0.1828$ is the effective mass ratio for a clamped circular plate,[12] a is the major modal radius, b is the minor modal radius, h_{drum} is the drumhead thickness, and ρ is the mass density of $NbSe_2$ ($6467\ \mathrm{kg\,m^{-3}}$).[13] For simplicity, $a \geq b$. The estimated ξ is reasonable for device B since the calculated ξ using finite element method[14] deviates only by 0.65%. Meanwhile, the effective force can be written as

$$F_{eff} = m_{eff} A_{eff} \tag{S15}$$

where A_{eff} is the effective acceleration (km/s^2) extracted from Eq. 3 of the main text.

To find the Young's modulus of elasticity of $NbSe_2$ E_Y in Table 2 of the main text, we first define the fundamental root of the frequency equation of a clamped elliptical plate with a small eccentricity $\epsilon = \sqrt{1 - (b/a)^2}$ as[15]

$$\beta = \left(3.1961 + 0.7991\epsilon^2 + 0.7892\epsilon^4\right) \tag{S16}$$

If $a = b$, then β represents the fundamental root of the frequency equation for a clamped

circular plate.[15] For elliptical plates, E_Y, assumed to be isotropic, can be approximated from the fundamental mode frequency of a clamped circular plate, with radius a and constant β from Eq. (S16)[15]

$$E_Y = 12(1-\nu^2)\rho\left(\frac{2\pi f_m a}{\beta^2 h_{drum}}\right)^2 \tag{S17}$$

where $\nu = 0.24$ is the Poisson ratio,[16] and f_m is the detected resonant frequency of the plate. For device A, an a/b ratio of 1.03 will lead to $\epsilon_A = 0.23$, $\beta_A^2 = 10.49$ and $E_Y = 123\,\text{GPa}$. For device B, an a/b ratio of 1.2 will lead to $\epsilon_B = 0.552$, $\beta_B^2 = 12.34$ and $E_Y = 148\,\text{GPa}$. The values presented in Table 2 in the main text is the average and standard deviation of the two values.

Details About Calibration of Nanomechanical Motion from the Setup

From the experimental setup shown in Fig. S1, we deduce the transduction coefficient $\alpha^{1/2}$ of our FP interferometer (in $\mu V_{pk}/\text{pm}$) as

$$\sqrt{\alpha} = \left|d\overline{R}_4(\lambda, h, s, \theta_{NA})/ds\right| G_{PD}(\lambda) T_{out}(\lambda) P_{in} \tag{S18}$$

Here, G_{PD} is the conversion gain of the photodetector (V/W), T_{out} is the transmittance of the output chain of the interferometer from point A to point B, and P_{in} is the input laser power used to probe our devices (in μW). The interferometer gain $S(\lambda_{probe})$ in our measurement is simply the product of $G_{PD}(\lambda = 532) = 8.42 \times 10^5$ V/W, and $T_{out} = 5.62 \times 10^{-3}$. The output chain includes all optical components covered by the light path from point A to point B in Fig. S1. The photodetector amplifies the reflected output power of the FP cavity, $T_{out} \times P_{in}$, and then multiply the product with G_{PD} to determine the output voltages. The discrepancies in the values of the transduction factors acquired from Brownian motion and our method, presented in Table 3 of the main manuscript, are attributed to unavoidable

scattering and absorption losses.

Table 4: Parameters Used to Estimate $\sqrt{\alpha_{MIA}}$ in Table 3 of the Main Manuscript

References	[12]	[17]	[18]
FP System	Vac-MoS_2-Vac-Si	Vac-Gr-Vac-Si	Vac-MoS_2-Vac-Si
s (nm)	290	385	290
P_{in} (μW)	670^a	800	300 (1L)^a<br>330 (3L)^a
G_{PD} (10^3 V/W)	16	16	16
T_{out} (10^{-3})	551	19	86
$\lvert d\overline{R}_4(\theta=0)/ds \rvert$ (10^{-6}/nm)	736.3^b	3523^c	470 (1L)^a<br>1210 (3L)^a
NA	0.5	0.6 [19]	0.5
$\gamma(NA)\times \lvert d\overline{R}_4(\theta=0)/ds \rvert$ (10^{-6}/nm)	387.2^b	2525^c	247 (1L)^b<br>636 (3L)^b

^{a} Inferred from Supporting Information of cited reference

^{b} Estimated using MIA with the refractive index database of MoS_2 [20]

^{c} Estimated using MIA with the refractive index database of graphite [6]

The estimated transduction factor and effective mass of the resonators provide hints on the resolvable Brownian Motion of the resonator. The Brownian motion of the nanomechanical resonator is expressed by its displacement spectral density, which is defined by [14]

$$S_z(f) = \frac{k_B T f_m}{2\pi^3 m_{eff} Q_m \left[\left(f^2 - f_m^2\right)^2 + \left(f f_m / Q_m\right)^2 \right]} \tag{S19}$$

where T is the resonator temperature (293.15 K), f_m is the fundamental mode frequency, Q_m is the quality factor of the resonator, m_{eff} is the effective mass of the resonator and k_B is the Boltzmann's constant (1.381×10^{-23} J/K). $S_z(f)$ is embedded in the power spectral density of the interferometric setup as expressed as [14]

$$S_{vv}(f) = S_{vv}^{w} + \alpha S_z \tag{S20}$$

where S_{vv}^{w} is the noise related to the detectors and measurement setup and is expressed in units of V^2/Hz. Assuming that the measured Q_m for the driven case provides the lower bound of the Q-factor for Brownian motion, the estimated power spectral density (PSD), and

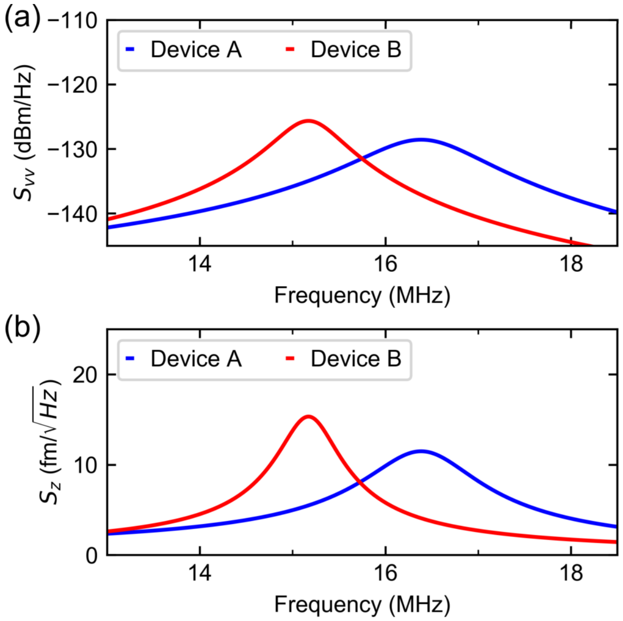

Figure S7: Estimating Brownian Motion of the $NbSe_2$ the circular (Device A) and elliptical devices (Device B). (a) Estimated Power Spectral Density S_{vv} (in dBm/Hz) of device A and device B. (b) Displacement power spectral density S_z of both device A and device B.

Brownian motion of the resonator should resemble Figs. S7(a-b), with the PSD expressed in units of dBm/Hz for clarity. Unfortunately, we do not see these features for the two devices due to the -110 dBm/Hz noise floor, which we attribute to the vibration noise level of our optical setup. Improving the fabricated device Q_m by a factor of 100, reducing the resonator thickness by one order lower, and limiting the system noise floor to that of our photodetector should help resolve the Brownian motion experimentally.

References

(1) Baumeister, P. W. *Optical Coating Technology*; SPIE - The International Society for Optical Engineering: Bellingham, Washington USA, 2004; Vol. PM137.

(2) Orfanidis, S. *Electromagnetic Waves and Antennas*; Rutgers University, 2016.

(3) Byrnes, S. J. Multilayer Optical Calculations. *arXiv:1603.02720v5* **2016**,

(4) Mak, K. F.; Sfeir, M. Y.; Wu, Y.; Lui, C. H.; Misewich, J. A.; Heinz, T. F. Measurement of the Optical Conductivity of Graphene. *Phys. Rev. Lett.* **2008**, *101*, 196405.

(5) Hill, H. M.; Rigosi, A. F.; Krylyuk, S.; Tian, J.; Nguyen, N. V.; Davydov, A. V.; Newell, D. B.; Walker, A. R. H. Comprehensive Optical Characterization of Atomically Thin $NbSe_2$. *Phys. Rev. B* **2018**, *98*, 165109.

(6) Palik, E. D., Ed. *Handbook of Optical Constants of Solids*; Academic Press: Burlington, 1997.

(7) GMBH, A. *E-BEAM RESISTS*; 2016; Accessed: 2020-05-05.

(8) Wang, Z.; Feng, P. X. Interferometric Motion Detection in Atomic Layer 2D Nanostructures: Visualizing Signal Transduction Efficiency and Optimization Pathways. *Sci. Rep.* **2016**, *6*, 28923.

(9) Darvishzadeh, A.; Alharbi, N.; Mosavi, A.; Gorji, N. E. Modeling the Strain Impact on Refractive Index and Optical Transmission Rate. *Physica B: Condensed Matter* **2018**, *543*, 14–17.

(10) Davidovikj, D. Two-dimensional membranes in motion. Ph.D. thesis, Delft University of Technology, 2017.

(11) Schneider, P.-I.; Garcia Santiago, X.; Soltwisch, V.; Hammerschmidt, M.; Burger, S.; Rockstuhl, C. Benchmarking Five Global Optimization Approaches for Nano-Optical Shape Optimization and Parameter Reconstruction. *ACS Photonics* **2019**, *6*, 2726–2733.

(12) Lee, J.; Wang, Z.; He, K.; Shan, J.; Feng, P. X.-L. High Frequency MoS_2 Nanomechanical Resonators. *ACS Nano* **2013**, *7*, 6086–6091.

(13) Meerschaut, A.; Deudon, C. Crystal Structure Studies of the $3R-NbSe_{1.09}S_2$ and the

$2H-NbSe_2$ Compounds: Correlation between Nonstoichiometry and Stacking Type (=Polytypism). *Mater. Res. Bull.* **2001**, *36*, 1721–1727.

(14) Hauer, B.; Doolin, C.; Beach, K.; Davis, J. A General Procedure for Thermomechanical Calibration of Nano/Micro-Mechanical Resonators. *Ann. Phys.* **2013**, *339*, 181 – 207.

(15) Shibaoka, Y. On the Transverse Vibration of an Elliptic Plate with Clamped Edges. *J. Phys. Soc. Japan* **1956**, *11*, 797 – 803.

(16) Project, T. M. Materials Data on NbSe2 by Materials Project. 2020; https://www.osti.gov/biblio/1197331.

(17) Davidovikj, D.; Slim, J. J.; Cartamil-Bueno, S. J.; van der Zant, H. S.; Steeneken, P. G.; Venstra, W. J. Visualizing the Motion of Graphene Nanodrums. *Nano Lett.* **2016**, *16*, 2768–2773.

(18) Lee, J.; Wang, Z.; He, K.; Yang, R.; Shan, J.; Feng, P. X.-L. Electrically Tunable Single- and Few-Layer MoS_2 Nanoelectromechanical Systems with Broad Dynamic Range. *Sci. Adv.* **2018**, *4*, eaao6653.

(19) Castellanos-Gomez, A.; van Leeuwen, R.; Buscema, M.; van der Zant, H. S.; Steele, G. A.; Venstra, W. J. Single-layer MoS(2) mechanical resonators. *Adv. Mater.* **2013**, *25*, 6719–23.

(20) Beal, H. P., A. R.; Hughes Kramers-Kronig Analysis of the Reflectivity Spectra of 2H-MoS_2, 2H-$MoSe_2$ and 2H-$MoTe_2$. *J. Phys. C; Solid State Phys.* **1979**, *12*, 881–890.
